# Is Natural Ventilation a Useful Tool to Prevent the Airborne Spread of TB?

**DOI:** 10.1371/journal.pmed.0040077

**Published:** 2007-02-27

**Authors:** Peter Wilson

## Abstract

Wilson discusses a new study in*PLoS Medicine* that examined the effect of natural ventilation in eight hospitals in Lima, Peru upon risks of TB transmission.

Airborne transmission of infections such as tuberculosis (TB) can be a major problem in health care establishments. Health care workers infected with TB can be unwitting disseminators of the infection, and expensive retrospective screening (of other health workers and patients) is required when the diagnosis is made. In the past, the benefits of natural ventilation in the treatment of TB were implicit in the design of the sanatoria wards (Figure 1). More recently, expensive negative pressure facilities have been installed to accommodate patients with TB, particularly following outbreaks of TB among patients with HIV/AIDS [1].

**Figure 1:**
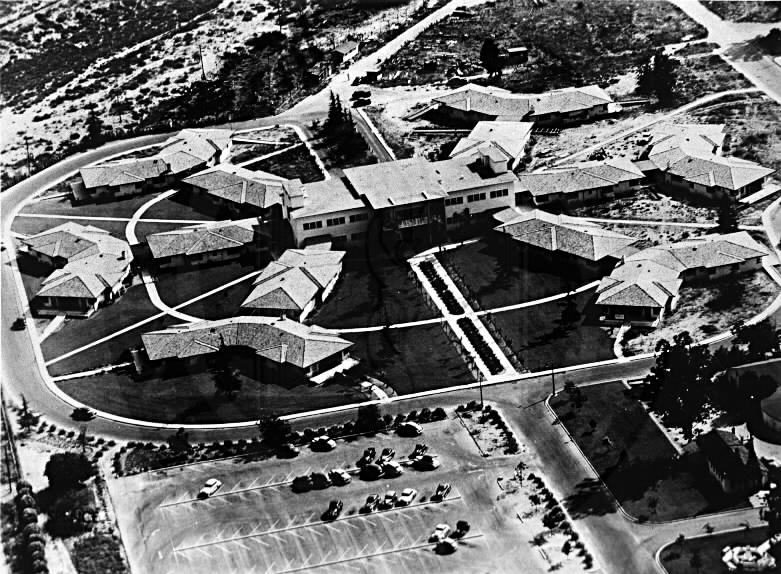
Los Angeles Sanatorium, Duarte, California, United States (Photo: City of Hope).

Room air should be changed 10–12 times every hour to ensure sufficient dilution of the bacterial load. Actual room air changes often fall below this level because of deficiencies in the facility and lack of maintenance. Respiratory masks provide protection, but are often inadequately fitted to the face and are not used before diagnosis is reached. Communal areas such as waiting rooms do not even approach these criteria and remain a risk for transmission. In a single incident of a mother and child with undiagnosed TB in a ward over several weeks, 6.7% of patients and 1.9% of pediatric health care workers had tuberculin skin test conversions [2]. Screening was required in 16 pediatric patients and 293 health care workers.

In many poorer countries, negative pressure ventilation may not be installed or monitored adequately [3]. Current recommendations are directed at the importance of early diagnosis and treatment in these areas, yet practice can often be less than ideal. In India facilities may be crowded and poorly ventilated, and they may lack infection control procedures [4]. The trainee medical staff is at particularly high risk of acquisition of TB [5]. However, many cases of nosocomial transmission are avoidable since the need for respiratory isolation can be accurately predicted on the basis of clinical and chest radiographic findings [6].

## A New Study Based in Peru

In a new study in *PLoS Medicine*, Roderick Escombe and colleagues examined the effect of natural ventilation in eight hospitals in Lima, Peru [7]. The study included 70 naturally ventilated clinical rooms, in five older and three newer hospitals, including respiratory and medical wards, clinics, and waiting rooms. The comparators were 12 negatively pressured rooms built in the last six years. A carbon dioxide tracer gas was used to establish the number of air changes in various sizes of rooms, with and without open windows and doors. Infection risk for TB was predicted using a model of airborne infection risk (the Wells-Riley equation). Larger rooms have higher absolute ventilation for a given number of air changes. To determine absolute ventilation, mechanically ventilated rooms were generally assumed to deliver 12 air changes per hour, but this may not be the case in many poorly monitored settings.

Natural ventilation provided 28 air changes per hour compared to 12 in negative pressure rooms. The high ceilings and large windows of old buildings allowed 40 air changes per hour. Rooms with open windows showed a 6-fold greater absolute ventilation than that calculated for mechanically ventilated rooms. In fact, poor maintenance of ventilation plants meant that only half the recommended air changes were delivered. The model of infection risk suggested a 39% infection rate in 24 hours of exposure to open tuberculosis in negative pressure rooms and 97% in closed unventilated rooms (13 infectious quanta per hour). In contrast, the rate with natural ventilation was 33% in newer buildings and 11% in older buildings.

## Implications of the Study

The study is a novel attempt to bring some scientific scrutiny to old practices. However, the Wells-Riley model that the authors used has some limitations. It assumes a steady state, and that one event leads to infection, while disregarding the effect of proximity to an infected case. Removal of infectious particles from the air by other processes including gravity is not considered.

The risk of airborne spread was far lower in rooms with open windows than the expensive mechanically ventilated rooms. For obvious reasons, low -cost natural ventilation seems to be a better option than negative pressure ventilation in tropical countries. The main drawback is possible contamination of adjacent areas when airflow is inward. These findings throw the old practice of situating TB wards at the top of a building and open to the elements in a new light. Dilution of the air is sufficient to keep the risk low. However, natural ventilation is not an easy solution for patients in countries where winters are cold. Further work is needed by the engineers to design cheap and reliable ventilation for rooms that promote rather than prevent passage of air yet allow thermal control. The current practice of sealing in the local environment is probably the wrong route for hospital wards.

## Abbreviations

TB: tuberculosis
